# Antioxidant and anti-inflammatory effects of Astragalus polysaccharide on EA.hy926 cells

**DOI:** 10.3892/etm.2013.1074

**Published:** 2013-04-23

**Authors:** WEI MIN HUANG, YONG QI LIANG, LI JUN TANG, YUE DING, XIAO HONG WANG

**Affiliations:** Department of Neonatology, Nanfang Hospital, Southern Medical University, Guangzhou, Guangdong 510515, P.R. China

**Keywords:** bronchopulmonary dysplasia, oxidative stress, inflammatory response, astragalus polysaccharide

## Abstract

The aim of this study was to explore the role of astragalus polysaccharide (APS) in the pathogenesis of bronchopulmonary dysplasia (BPD) in preterm children using an established BPD cell model. EA.hy926 cell cultures were divided into three groups: the air group as the blank control, the hyperoxia group as the experimental control and the APS group (2.5 mg/ml). The production of superoxide dismutase (SOD), malondialdehyde (MDA) and reactive oxygen species (ROS) were analyzed by biochemical assays. Real-time reverse transcription-polymerase chain reaction (RT-PCR) and western blotting were used to detect the RNA and protein expression levels of inflammatory cytokines, including interleukin (IL)-8, intercellular adhesion molecule 1 (ICAM-1) and nuclear factor (NF)-κB p65. Compared with the hyperoxia group, the ROS and MDA levels of the APS group were significantly reduced. By contrast, SOD production was significantly increased. The expression of IL-8, ICAM-1 and NF-κB p65 in the APS group was downregulated. APS acts as an antioxidant by stimulating SOD production while inhibiting lipid peroxidation in the EA.hy926 cells. Furthermore, this study demonstrated that APS retards the inflammatory response, as shown by the reduced expression of NF-κB p65, IL-8 and ICAM-1 when APS was added.

## Introduction

Bronchopulmonary dysplasia (BPD) is the most common complication of preterm children who accept long-term mechanical ventilation or oxygen therapy. BPD seriously affects the survival of premature children and quality of life (1). While the etiology and pathogenesis of BPD remain complex, lines of evidence indicate that oxidative stress and inflammatory responses play crucial roles in the disease development (2–4). Clinical trials of antioxidant and anti-inflammatory agents have been conducted. One of the most well-investigated types of agent is glucocorticoids. Glucocorticoids have been shown to reduce the incidence of BPD by inhibiting the inflammatory response through the production of antioxidant enzymes, improving pulmonary edema and fibrosis (5). However, the side-effects of glucocorticoids, including increased mortality, growth retardation, cerebral palsy, hyperglycemia, intestinal perforation and infection, limit their long-term use (6,7). Therefore, the identification of more efficient and safer replacements for BPD treatment is required.

Astragalus polysaccharide (APS) is a traditional Chinese medicine that has been studied in depth and is widely used in clinics. APS is one of the most important natural active materials due to its multi-targeting biological activities, including antioxidant, radical scavenging and anti-inflammatory activity and immune regulation (8). Gradient and alcohol precipitation, chromatography, ultrafiltration, microfiltration and other modern pharmaceutical technology are used in the preparation of APS, so that the effective ingredients are accurately separated and purified; the purity is 98%. The antioxidative and anti-inflammatory effects of APS have been well-recognized in previous studies (9–11). To the best of our knowledge, studies concerning APS in BPD are rare. Whether APS prevents or rescues the development of BPD by antioxidant and/or anti-inflammatory actions remains unclear due to the lack of well-built models and clinical trials.

The EA.hy926 cell line is an immortalized human umbilical vein endothelial cell (HUVEC) line, derived from the fusion of HUVECs and lung adenocarcinoma cells. The structural characteristics and functions are similar to lung adenocarcinoma cells and these alveolar endothelial cells are used to construct the alveoli *in vitro* mouse model (12). EA.hy926 cells have been widely applied in the study of leukocyte adhesion to endothelial cells, oxidative stress and protein expression (13–18).

In the current study, we aimed to explore the role of APS in preterm children with BPD and its mechanism of action by establishing an *in vitro* cell model of BPD. Our findings indicated that APS mitigates the cell damage induced by oxidative stress and inhibits the inflammatory response by downregulating the production of reactive oxygen species (ROS) and malondialdehyde (MDA) and the expression of nuclear factor (NF)-κB, as well as increasing the levels of superoxide dismutase (SOD).

## Materials and methods

### Reagents and materials

EA.hy926 cells were commercially available from the Shanghai Institute Cell Bank (Shanghai, China). Fetal bovine serum (FBS; Cat# 16000-044) and high glucose Dulbecco’s modified Eagle medium (DMEM; Cat# C11995) were purchased from Gibco (Carlsbad, CA, USA). APS was purchased from Tianjin Cinorch Pharmaceutical Co., Ltd. (Tianjin, China). The triple gas mixture (70% O_2_, 5% CO_2_ and 25% N2) was provided by Nanfang Hospital Oxygen Center (Guangzhou, China). The SOD and MDA test kits were provided by Nanjing Jiancheng Bioengineering Institute (Nanjing, China). The ROS kit (C1300) was purchased from Beijing Applygen Technologies Inc. (Beijing, China). Anti-intercellular adhesion molecule 1 (ICAM-1; Cat# 3518-1), anti-interleukin (IL)-8 (Cat# 3482-1) and anti-NF-κB p65 (Cat# 2229-1) were purchased from Epitomics Inc. (Burlingame, CA, USA).

### Cell culture

EA.hy926 cells were cultured in high glucose DMEM containing 10% FBS at 5% CO_2_ in a 37°C incubator. Then, EA.hy926 cells were divided into three groups: the air group, the hyperoxia group and the APS group (2.5 mg/ml). The air group were cultured for 24, 36 and 48 h in a 5% CO_2_, 37°C incubator. The hyperoxia group and the APS group were cultured for 24, 36 and 48 h in the 37°C triple gas mixture.

### Assessment of ROS

Following removal of the culture medium, the cells were washed twice with phosphate-buffered saline (PBS). Then, 1 ml H_2_O_2_ (100 *μ*M) was added to the cells of the air group as the positive control and 1 ml 2′,7′-dichlorofluorescein-diacetate (DCFH-DA; 10 *μ*M) was added to the cells of the hyperoxia and APS groups. The cells were then incubated for 30 min at 37°C. Following pipetting of the mixture from the petri dish, the cells were washed twice with PBS and visualized under a fluorescence microscope (IX71; Olympus, Tokyo, Japan). The fluorescence was measured using an MD5 microplate reader (SpectraMax M5; Molecular Devices Company, Sunnyvale, CA, USA).

### Measurement of SOD and MDA

Cells were sonicated by ultra-sound and mixed according to the manufacturer’s instructions. The SOD mixture was incubated in water at 37°C for 40 min and then cooled to room temperature for 10 min after adding chromogenic reagent. Absorbance at a wavelength of 550 nm was measured by the MD5 microplate reader. The MDA mixture was boiled for 80 min and then cooled by flowing water. Absorbance at a wavelength of 532 nm was measured by the MD5 microplate reader.

### Western blotting

Total protein was extracted from the cells according to the manufacturer’s instructions (Cat# P0027; Beyotime Institute of Biotechnology, Jiangsu, China). Then, 20 *μ*g proteins and ladder were fractionalized using sodium dodecyl sulfate-polyacrylamide gel electrophoresis (SDS-PAGE) and electrophoretically transferred and blotted onto a nitrocellulose membrane. Non-specific binding was blocked by 1 h incubation of the membranes with blocking buffer [(5% non-fat dry milk and 0.05% Tween-20 in Tris-buffered saline (TBS)]. The blots were then probed overnight at 4–6°C with primary mouse anti-β-actin (1:5,000; Novus Biologicals, Littleton, CO, USA), anti-ICAM-1 (1:1,000), anti-IL-8 (1:1,000) or anti-NF-κB p65 (1:100,000) in blocking buffer. After three washes, the membranes were incubated with horseradish peroxidase-conjugated goat anti-mouse IgG. The blots were developed using light-emitting liquid (Cat# WBKLS0100; Millipore, Billerica, MA, USA) and quantified using an Odyssey instrument (Nanfang Hospital Clinical Trial Center, Guangzhou, China) (19).

### RT-PCR

The total RNA of cells was extracted using TRIzol reagent (Cat# D9108A; Takara Biotechnology Co., Ltd., Dalian, China) and cDNA was synthesized according to the instructions from the PrimeScript^®^ RT reagent kit with gDNA Eraser (Perfect Real Time; Cat# DRR047S; Takara Biotechnology Co., Ltd.). RT-PCR primers were as follows: GAPDH, forward: 5′-agaaggctggggctcatttg-3′ and reverse: 5′-aggggccatccacagtcttc-3′; IL-8, forward: 5′-tagcaaaattga ggccaagg-3′ and reverse: 5′-aaaccaaggcacagtggaac-3′; and ICAM-1, forward: 5′-ggctggagctgtttgagaac-3′ and reverse: 5′-actgtggggttcaacctctg-3′. The reaction system was prepared according to the instructions of the SYBR^®^ Premix Ex Taq™ kit (Perfect Real time; Cat# DRR420A; Takara Biotechnology Co., Ltd.) and then DNA was amplified as follows: 95°C for 10 min; then 95°C for 15 sec, 61°C for 15 sec and 72°C for 15 sec for 40 cycles; and finally 95°C for 1 min, 55°C for 30 sec and 95°C for 30 sec.

### Statistical analysis

Data are expressed as mean ± standard deviation and analyzed using SPSS 13.0 (SPSS, Inc., Chicago, IL, USA). Statistical comparisons between groups were performed by factorial analyses. P<0.05 was considered to indicate a statistically significant difference.

## Results

### SOD and MDA assessment

**Figure 1.**
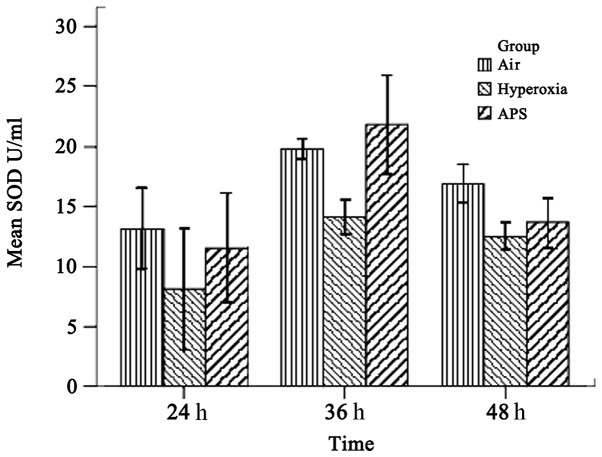
SOD levels in the air, hyperoxia and APS groups. The SOD level of the hyperoxia group was lower than those in the air and APS groups after culture for 24 and 36 h; however, there was no significant difference between the SOD levels of the air and APS groups (P>0.05). At 48 h, there was no statistically significant difference between the hyperoxia and APS groups (P>0.05); however, the content of SOD in the air group was significantly higher. The SOD level of the APS group at 36 h was significantly higher compared with that at 24 h (F=28.845, P<0.01). SOD, superoxide dismutase; APS, astragalus polysaccharides.

**Figure 2.**
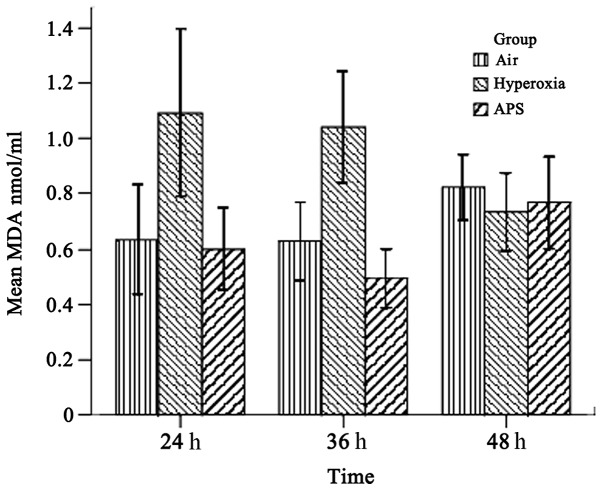
MDA levels in the air, hyperoxia and APS groups. The MDA level of the hyperoxia group was significantly higher than those in the air and APS groups at 24 and 36 h; however, there was no significant difference between the air and APS groups (P>0.05). The comparison between the three groups at 48 h was not statistically significant. MDA, malonidaldehyde; APS, astragalus polysaccharides.

As shown in Figs. 1 and 2, the results demonstrated that the SOD and MDA levels of the three groups were significantly different (SOD, F=15.842, P<0.01; MDA, F=21.950, P<0.01). The average SOD level of the hyperoxia group (11.586±3.955 U/ml) was lower than those of the air group (16.609±3.472 U/ml) and the APS group (15.679±5.737 U/ml). By contrast, the MDA level of the hyperoxia group (0.950±0.270 nmol/ml) was lower than those of the air group (0.682±0.170 nmol/ml) and the APS group (0.622±0.180 nmol/ml). Furthermore, time course analysis demonstrated significant differences when the cells were cultured for 24, 36 and 48 h, respectively (F=32.555, P<0.01). It is clear that APS induces the production of SOD in EA.hy926 cells most significantly at 36 h (21.818±4.054 U/ml), while there were no significant differences in the MDA level among the three time points (F=0.662, P=0.520). These data suggest that APS induces the secretion of SOD by EA.hy926 cells and blocks MDA generation at the same time.

### ROS analysis

**Figure 3.**
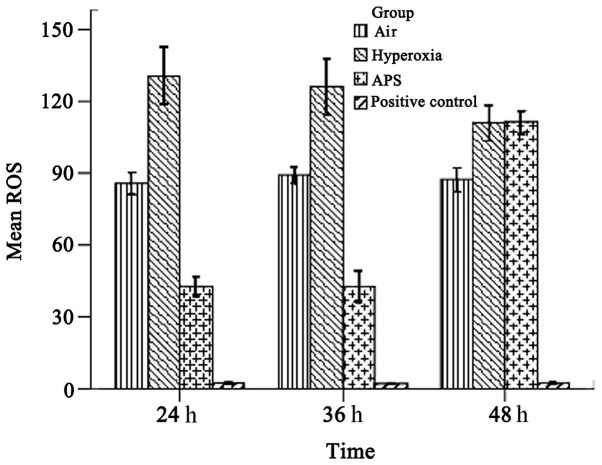
ROS levels in the air, hyperoxia and APS groups. The ROS level of the hyperoxia group was significantly higher than those of the air and APS groups at 24 and 36 h while the ROS level of the APS group was significantly lower than that of the air group (P<0.01). The ROS level of the air group was significantly lower compared with those of the hyperoxia and APS groups at 48 h, while the difference between the hyperoxia and APS groups was not statistically significant (P>0.05). The ROS level of the APS group at 48 h was significantly higher compared with the ROS level at 24 and 36 h (F=675.247, P<0.01). ROS, reactive oxygen species; APS, astragalus polysaccharides.

As shown in Fig. 3, the average ROS levels were 87.465±4.365, 122.370±7.253, and 65.632±4.046 for the air, hyperoxia and APS groups, respectively; there were significant differences among the groups (F=955.604, P<0.01). The results demonstrate that the ROS level of the EA.hy926 cells was reduced by treatment with APS.

### IL-8 and ICAM-1 transcription

**Figure 4.**
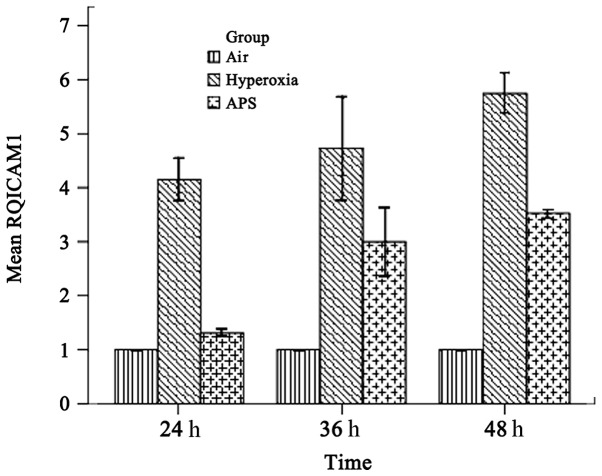
Inhibition of ICAM-1 transcription at different time points by APS in EA.hy926 cells. The results demonstrated that the ICAM-1 gene expression levels of the hyperoxia group at 24, 36 and 48 h were significantly higher compared with those of the APS group. The ICAM-1 gene expression levels of the hyperoxia group at 24, 36 and 48 h were 4-, 1.58- and 1.63-fold higher than the ICAM-1 gene expression level of the APS group at the same time point. The ICAM-1 expression levels at 24, 36 and 48 h were not statistically significantly different (F=1.947, P=0.159). ICAM-1, intercellular adhesion molecule 1; APS, astragalus polysaccharides; RQ, relative quantity.

**Figure 5.**
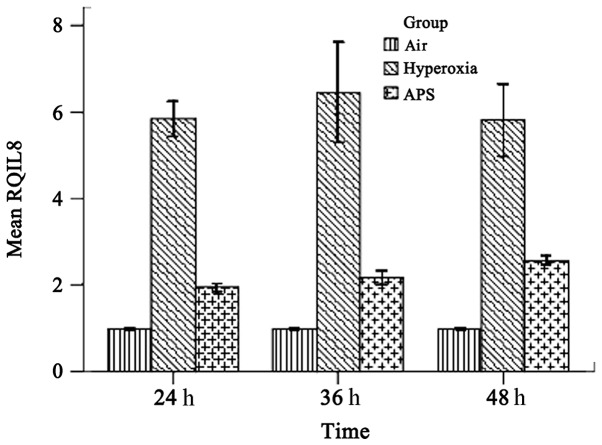
Inhibition of IL-8 transcription at different time points by APS in EA.hy926 cells. The results demonstrated that the IL-8 gene expression levels in the hyperoxia group at 24, 36 and 48 h were significantly higher compared with those of the APS group. The IL-8 gene expression levels of the hyperoxia group at 24 and 36 h were 3-fold higher than the IL-8 gene expression level of the APS group. The IL-8 gene expression level of the hyperoxia group at 48 h was double the level of the APS group. The IL-8 expression levels at 24, 36 and 48 h were not statistically significantly different (F=0.233, P=0.794). IL, interleukin; APS, astragalus polysaccharides; RQ, relative quantity.

As shown in Figs. 4 and 5, the results demonstrated that IL-8 and ICAM-1 RNA expression was downregulated by APS, at the average levels of 2.227±0.289 and 2.611±1.052 respectively, compared with 6.033±0.808 and 4.880±0.891 in the hyperoxia group. The expression levels of IL-8 and ICAM-1 in the APS group were significantly lower than the respective levels in the hyperoxia group (IL-8, F=205.373, P<0.01; ICAM-1, F=158.926, P<0.01).

### Western blotting

The protein expression levels of NF-κB p65, ICAM-1 and IL-8 in the APS group were significantly lower than those in the hyperoxia group at each time point. However, NF-κB p65 and IL-8 expression levels were not significantly altered over different time points.

## Discussion

Oxidative stress and the inflammatory response are two important events during BPD development. SOD removes the superoxide anion radical in order to protect cells from damage (20). An imbalance between SOD and ROS directly affects MDA production and oxidative stress injury. In addition, cytokines play important roles in the lung inflammatory response. A variety of cytokines are involved in the occurrence of BPD, including IL-6, IL-8, IL-10, tumor necrosis factor (TNF)-β and ICAM-1. Therefore, infection leads to or worsens BPD severity due to the release of inflammatory stimuli. Airway epithelial cells and pulmonary capillary endothelial cells are damaged by inflammatory regulators, resulting in the release of regulatory factors of inflammation and chemokines, as well as infiltration of inflammatory cells. Neutrophils participate in the inflammatory response and produce cytokines, including IL-8, macrophage and inflammatory response protein-1 and TNF-β, which enhance vascular permeability, resulting in pulmonary interstitial, alveolar and airway edema (21). The current study identified that the ROS and MDA levels of the APS group were significantly reduced compared with those of the hyperoxia group (P<0.05), while the SOD content was increased compared with that in the hyperoxia group (P<0.05). The expression of IL-8, ICAM-1 and NF-κB p65 in the APS group was lower than in the hyperoxia group. These assays indicate that APS acts against oxidative stress injury and reduces the inflammatory response.

The oxidative stress response is regulated by an imbalance between ROS generation and antioxidant enzyme degradation of ROS (22). Under physiological circumstances, there is a delicate balance between human ROS generation and the antioxidant defense system. The balance is likely to be broken when the overexpression or inadequate clearance of ROS is out of the metabolic control of cells, resulting in oxidative stress injury (23,24). It is known that SOD contributes to scavenging ROS. The primary role of SOD is to translate highly reactive superoxide free radicals into hydrogen peroxide and water; hydrogen peroxide is then transformed into water by catalase, glutathione peroxidase and glutathione reductase. Oxidative stress injury results in lipid peroxidation and produces MDA. SOD scavenges oxygen free radicals to protect cells from damage; the level of its activity indirectly reflects the ability of the body to scavenge oxygen free radicals.

APS, as a potent antioxidant, effectively inhibits the generation of oxygen free radicals, preventing membrane lipid peroxidation and reduction of biofilm injury. APS ameliorates hypoxia and prevents reperfusion lung injury (25). *In vitro* tissue culture experiments indicate that APS stimulates the total antioxidant capacity of cells, as well as reduces the generation of oxygen free radicals and thus prevents rat lung epithelial cell injury. APS also protects the intestinal mucosa of rats with obstructive jaundice from oxidative stress damage, which may relate to increases in SOD levels and reductions in MDA levels (26). Li XT *et al* identified that APS promotes the wound healing of chronic ulcers, by a mechanism related to the reduced expression of inflammatory stimuli, wound lipid peroxidation and enhancement of SOD expression (27). The activity of SOD in dogs’ blood may be significantly improved by APS. APS increases the activity of superoxide dismutase, as well as reduces the content of lipid peroxide and the cell damage caused by free radicals (28). Our study identified that APS is able to prevent the production of ROS due to intracellular oxidative stress by increasing the expression of SOD in EA.hy926 cells and reducing the peroxidation of the cytoplasm, which protects cells from oxidative stress injury.

The inflammatory response plays a crucial role in the development of BPD. A variety of cytokines, including IL-6, IL-8, IL-10 and ICAM-1, are considered to participate in lung inflammation, which is closely related to the development of BPD. NF-κB is one of the transcription factors regulating the expression of inflammatory cytokines. NF-κB upregulates the expression of a variety of inflammatory cytokines, which induce inflammation and then promote the development of BPD. NF-κB is a heterodimer complex, composed of p65/p50. NF-κB binds to the inhibitory-type IκB protein in the cytoplasm. When endothelial cell damage occurs, IκB is phosphorylated and degraded. Phosphorylated NF-κB translocates to the nucleus, binds to the nucleotide sequence of the κB domain and regulates the transcription of a variety of inflammatory cytokines and adhesion molecules (29,30). The activation of NF-κB leads to the overexpression of inflammation-related factors and causes a significant inflammatory response. In return, the increased inflammatory mediators and cytokines further activate NF-κB, amplifying the initial inflammatory signals. The activation of NF-κB stimulates endothelial cells to release IL-8. IL-8 is a potent neutrophil chemotactic factor that promotes and prolongs the inflammatory response (31). Wu *et al* identified that NF-κB also induces the production and release of ICAM-1 (32), which dilates blood vessels, causes the migration of inflammatory cells and induces the release of cytokines and chemokines into adherent tissues. A previous study demonstrated that the expression of VCAM-1, ICAM-1 and NF-κB mRNA in cardiac microvascular endothelial cells in reperfusion injury is suppressed by APS (33). In a lipopolysaccharide (LPS)-induced inflammatory response, the generation of TNF-α and IL-8 was inhibited by APS, which may play a role in preventing inflammation (34). In osteoarthritis, APS is reported to reduce the inflammatory response of synovial cells and the generation of apoptosis (35). The current study identified that APS inhibits the activation of NF-κB p65, thereby reducing the expression of ICAM-1 and IL-8 and the inflammatory response.

Oxidative stress and inflammatory responses affect and promote each other. Inflammatory cells release ROS and are involved in oxidative stress. However, ROS-induced oxidative stress activates NF-κB p65 signaling and promotes the expression of pro-inflammatory cytokine genes and the chemotaxis of inflammatory cells, including neutrophils (36). Additional oxidative stress may enhance the chemotaxis of neutrophils and macrophages and stimulate cell adhesion molecule expression, inducing the inflammatory response. The mechanisms by which APS inhibits the activation of NF-κB p65 may be: i) APS has a direct anti-inflammatory role by inhibiting the expression of NF-κB p65 directly; and ii) APS eliminates oxygen free radicals, which is the main stimulant of NF-κB p65 activation. Higher concentrations of APS have an improved scavenging effect on superoxide anion free radicals and lipid free radicals, with the concentration increasing as the scavenging effect increases.

In summary, oxidative stress and inflammation are two important pathological events of BPD, independently or interactively. Lipid peroxidation of EA.hy926 cells may be reduced by APS, which removes the oxygen free radicals within the cells as an antioxidant. In addition, the activation of NF-κB p65 may be effectively blocked and the expression of cytokines, including IL-8 and ICAM-1, may be reduced by APS, which inhibits the inflammatory response. This study was limited to *in vitro* experiments. The mechanisms of the antioxidant and anti-inflammatory effects of APS in the protection against BPD pathogenesis require further validation *in vivo*.
